# Commentary on “Force-field functor theory: classical force-fields which reproduce equilibrium quantum distributions”

**DOI:** 10.3389/fchem.2013.00034

**Published:** 2013-12-24

**Authors:** Ruggero Vaia

**Affiliations:** Istituto dei Sistemi Complessi - Consiglio Nazionale delle Ricerche and Istituto Nazionale di Fisica Nucleare - Sezione di FirenzeSesto Fiorentino, Italy

**Keywords:** quantum thermodynamics, effective classical potential, path integration, Monte Carlo simulations, force-field functor

A commentary on Force-field functor theory: classical force fields which reproduce equilibrium quantum distributions by Babbush, R., Parkhill, J., and Aspuru-Guzik, A. (2013). Front. Chem. 1:26. doi: 10.3389/fchem.2013.00026

This comment regards a recent paper by Babbush, Parkhill, and Aspuru-Guzik (henceforth BPA) (Babbush et al., 2013). The subject is the equilibrium thermodynamics of a system of many quantum particles with Hamiltonian $\hat{H}=\sum_{i\;=\;1}^{N}{\hat{p}}_{i}^{2}/(2m)+V(\hat{q})$, where $\hat{q}=\{{\hat{q}}_{i}\}$ and the commutator of the coordinate- and momentum operators is $[{\hat{q}}_{i},{\hat{p}}_{j}]=i\hbar \delta_{ij}$. In BPA it is correctly observed (see, e.g., Hillery et al., 1984; Cuccoli et al., 1995) that the coordinate distribution function at temperature *T* = β^−1^, namely

(1) $$\begin{array}{l} \eta (q)=\frac{1}{Z}\langle q|e^{-\beta \hat{H}}|q\rangle \\ \;\equiv \frac{1}{Z}{\left(\frac{m}{2\pi \hbar^{2}\beta }\right)}^{N/2}e^{-\beta W(q)}, \end{array}$$

can be used to define the *effective* potential *W*(*q*) (the kinetic part of the partition function *Z* is omitted in BPA) in terms of which the exact quantum equilibrium average of any operator $O(\hat{q})$ takes the classical form of a configuration integral,

(2) $$\begin{array}{l} \langle O(\hat{q})\rangle =\int dq\langle q|O(\hat{q})e^{-\beta \hat{H}}|q\rangle \\ \;=\frac{1}{Z}\text{​}{\left(\text{​}\frac{m}{2\pi \hbar^{2}\beta }\text{​}\right)}^{N/2}\text{​​​}\int \text{​}\text{​}dq\;O(q)\;e^{-\beta W(q)}. \end{array}$$

It is convincingly proven in BPA that the mapping η(*q*) ↔ *W*(*q*) (i.e., the exponential function) is bijective, as well as *V*(*q*) ↔ η(*q*), and then it follows that *V*(*q*) ↔ *W*(*q*) is one-to-one. Furthermore, it is correctly pointed out that the Giachetti–Tognetti–Feynman–Kleinert (Giachetti and Tognetti, 1985; Feynman and Kleinert, 1986) (GTFK) effective potential *V*_eff_(*q*) differs from *W*(*q*). Indeed, *V*_eff_(*q*), which accounts *exactly* for any quadratic potential, entails that for approximating $\langle O(\hat{q})\rangle$ one has to include a further Gaussian average accounting for purely-quantum fluctuations, as shown, e.g., in Vaia and Tognetti (1990); Cuccoli et al. (1995): there *W*(*q*) is also introduced and dubbed the *local* effective potential. However, in BPA it is not shown that Equation (29), the paper's main result obtained as the Jensen's approximation *W*(*q*) ≈ *W*_BPA_(*q*) to the exact formula (26), can be calculated explicitly as

(3) $$\begin{array}{l} W_{\text{BPA}}(q)=V(q)-\frac{1}{\beta }\langle U[r(\tau )]\rangle \\ \;=\langle \int_{0}^{\beta \hbar }\frac{d\tau }{\beta \hbar }\;V[r(\tau )]\rangle \\ \;=\int \text{​}\text{​}\frac{d^{N}\xi }{{\left(2\pi \text{​}\sigma^{2}\right)}^{\frac{N}{2}}}V(q+\xi )\;e^{-\frac{\xi^{2}}{2\sigma^{2}}},\;\;\; \end{array}$$

i.e., the convolution between the potential *V*(*q*) and a Gaussian with variance σ^2^ = β*ħ*^2^/(6*m*) proportional to the squared de-Broglie wavelength. This is in agreement with the Wigner series (Wigner, 1932) up to lowest order, but lacks the nonlinear contributions to it. How accurate is the approximation made in Equation (29) of BPA? One can estimate this by considering a single (*N* = 1) quantum harmonic oscillator, $V(\hat{q})=\kappa {\hat{q}}^{2}/2$, whose frequency is $\omega \equiv \sqrt{\kappa /m}$. Expanding *V*(*q* + ξ) in Equation (3) one finds

(4) $$\begin{array}{l} W_{\text{BPA}}(q)=\sum_{n=0}^{\infty }\frac{1}{n!}\;\frac{d^{2n}V(q)}{dq^{2n}}\;{\left(\frac{\beta \hbar^{2}}{12m}\right)}^{n}\\ \;=V(q)+\frac{\beta {\left(\hbar \omega \right)}^{2}}{12}\;, \end{array}$$

which does not improve upon the classical result using Equation (2). From the known density for the quantum harmonic oscillator the *exact functor* for the class of harmonic potentials can be easily derived:

(5) $$\begin{array}{l} V(q)=\frac{m\omega^{2}}{2}\;q^{2}\;\;\to \\ W(q)=\frac{1}{2\beta }\ln\frac{\sinh\beta \hbar \omega }{\beta \hbar \omega }+\frac{m\omega }{\beta \hbar }\;\tanh\frac{\beta \hbar \omega }{2}\;\;q^{2}. \end{array}$$

For a *linear* functor, this expression should be proportional to *m*ω^2^: evidently this is true only in the classical limit, β*ħ*ω « 1 or *T* » *ħ*ω, where Equation (4) is recovered. However, the mapping *V* → *W* can surely be *locally* linear, namely *V* + εδ*V* → *W* + εδ*W* with δ*W* independent of the small parameter ε. Hence, *W*_BPA_(*q*) is reliable only when the temperature overcomes the typical quantum energy scale *ħ*ω; for instance, taking ω^2^ ~ *V*″(*q*_m_)/*m* [*q*_m_ being the minimum of *V*(*q*)], a pair of hydrogen molecules has typically *ħ*ω ~ 10^2^ K (Vaia and Tognetti, 1990 and references therein) and using *W*_BPA_(*q*) would only be reliable at very high *T* » 10^2^ K, i.e., just in the classical limit. Such an approximation is indeed used in the high-*T* propagator of path-integral Monte Carlo algorithms in order to improve convergence in the Trotter number (Takahashi and Imada, 1984). Hence, the approximation (29) of BPA can “reproduce quantum distributions” just when these are almost classical.

On the other hand, the use of the *exact* effective *pair-potential*, rather than that obtained from Equation (29) of BPA, is a good starting point for treating a not too dense quantum fluid by means of a classical-like simulation, as shown in the last section of BPA and as noted by several authors (see, e.g., Thirumalai et al., 1984 and many references cited in BPA). At variance with the procedure of BPA, based on the heavy calculation of a (*locally*) “linear functor” at fixed *T*, it would be more practical to directly obtain the exact pair-potential *W*(*q*) for the chosen $V(\hat{q})$, a task that can easily be carried out at any *T*.
